# Severe recurrent anaphylaxis: investigating underlying mechanisms beyond IgE-mediated allergy — a case report

**DOI:** 10.3389/falgy.2026.1866446

**Published:** 2026-07-01

**Authors:** Agnieszka Gawlewicz-Mroczka, Ewelina Gacek, Natalia Zamorska, Krzysztof Sładek

**Affiliations:** 1 Jagiellonian University Medical College, Krakow, Poland; 2 Department of Pulmonology and Allergology at the University Hospital in Krakow, Krakow, Poland

**Keywords:** severe anaphylaxis, systemic mastocytosis, IgE-mediated allergy, histamine intolerance, defensin-related food allergy

## Abstract

Anaphylaxis is a sudden, life-threatening, and generalized allergic reaction that develops within minutes to hours following exposure to an allergen. Cutaneous, respiratory, and cardiovascular symptoms may occur in various combinations, and the severity of the reaction can vary, potentially leading to anaphylactic reactions, which, if untreated with epinephrine, may be fatal. Although anaphylaxis is most commonly caused by allergic mechanisms, cofactors and comorbidities can contribute to increased severity. We present the case of a 34-year-old man who repeatedly experienced severe anaphylactic reactions after consuming products containing defensins. Comprehensive diagnostic evaluation confirmed an IgE-dependent allergic reaction to inhalant defensins. Additionally, histamine intolerance was suspected, and suspicion of systemic mastocytosis (SM) was raised. This study aims to describe the diagnostic process and highlight the challenges in differentiating clinical symptoms arising from overlapping pathophysiological mechanisms in these conditions.

## Case report

A 34-year-old patient was admitted to the Department of Pulmonology and Allergology at the University Hospital in Kraków, Poland, for comprehensive assessment and diagnostic evaluation of severe anaphylactic episodes.

His medical history included three episodes of severe anaphylactic reactions. The first occurred in October 2022 after consuming a fruit mousse containing mango, apple, banana, and wheat. The second episode occurred one year after the first. The patient had consumed store-bought herring in cream sauce accompanied by a wheat roll. The most recent episode occurred in August 2025 during a barbecue gathering. After consuming a variety of foods, the last food consumed before the anaphylactic reaction was potatoes with herbs, the patient developed symptoms within 20–30 min, including a burning sensation of the face and tongue, dyspnea, tachycardia, facial swelling, conjunctival hyperemia, vomiting, and ultimately loss of consciousness. In the last of the three anaphylactic reactions, physical exercise was identified as a cofactor.

During the first episode the patient called emergency medical services and received intramuscular adrenaline, antihistamines and glucocorticosteroids intravenously, as well as intravenous fluids. During the second episode, the patient self-administered adrenaline using an auto-injector, and in the third episode, it was administered by the patient's wife. In all instances, the patient was hospitalized in the emergency department (ED). Blood samples for tryptase measurement were not collected during any of the episodes. During each episode, the patient was observed in the emergency department for around 5 h and was never transferred to an inpatient ward following the anaphylactic reaction.

**Figure 1:**
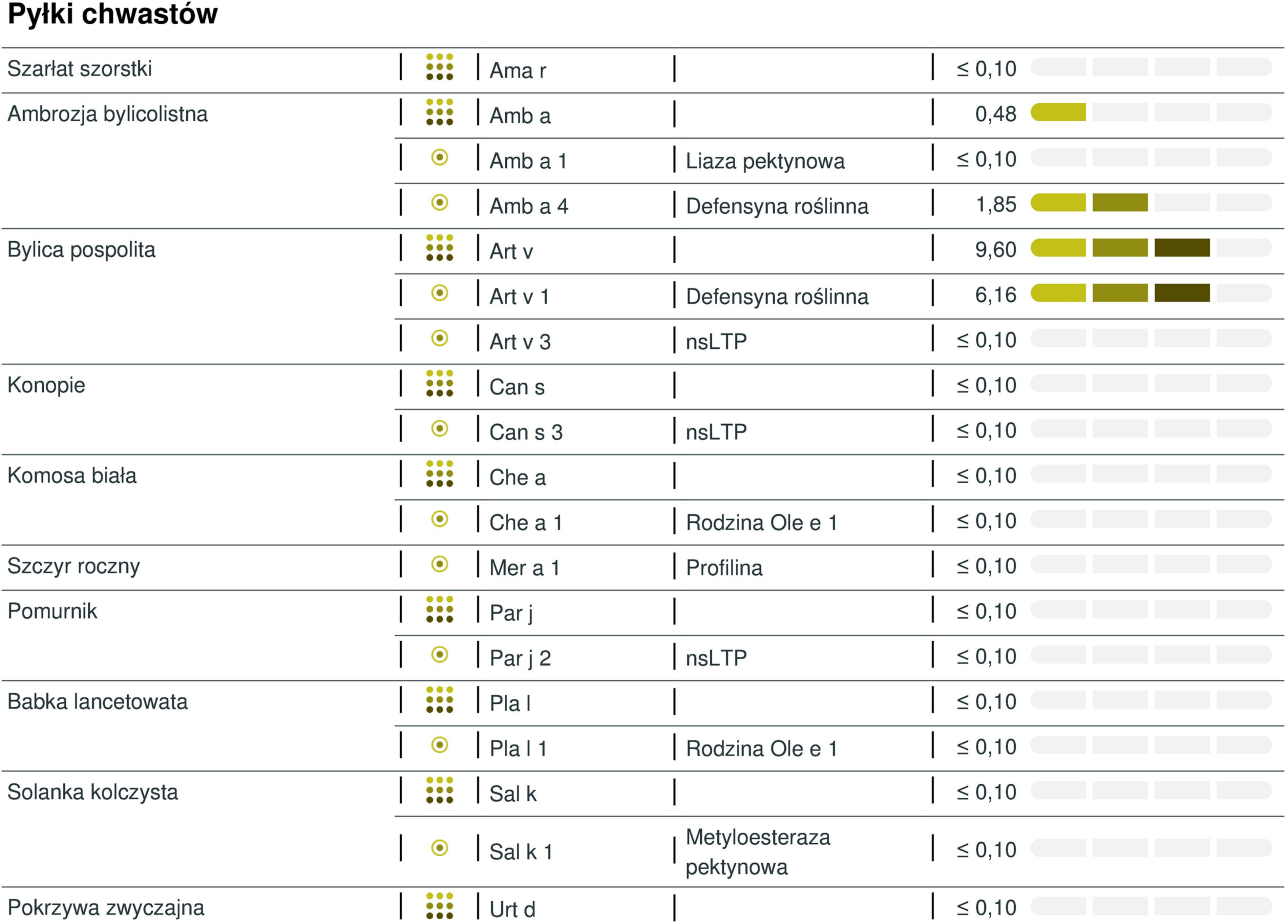
Component-resolved diagnostic results obtained using the ALEX^2^ multiplex platform. Positive sIgE levels for plant defensins (Amb a 4 and Art v 1).

**Figure 2:**
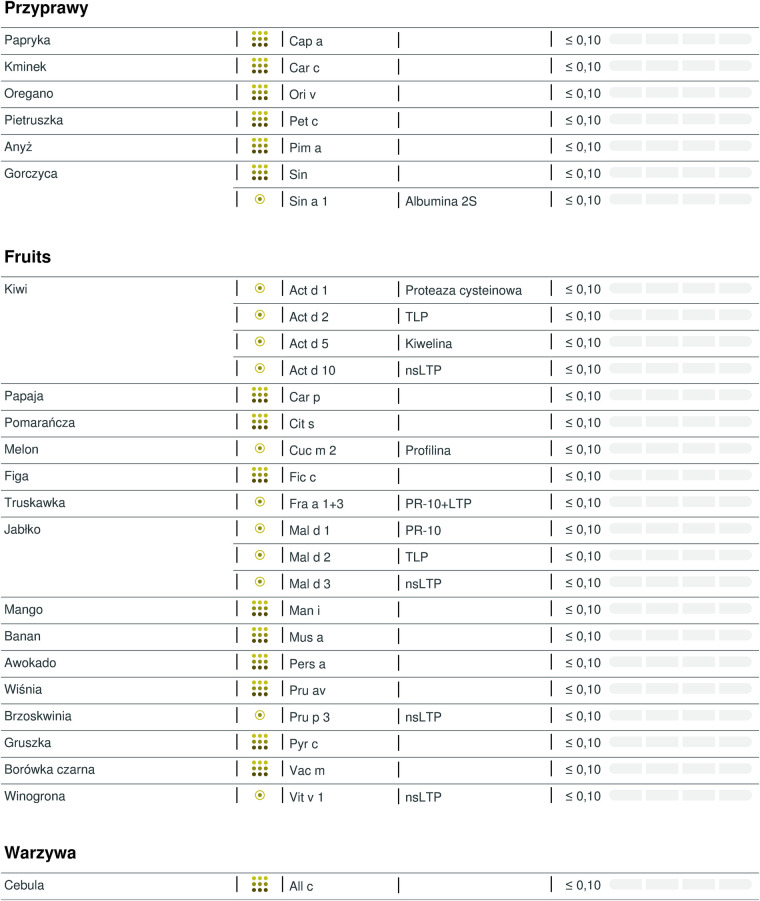
Component-resolved diagnostic results obtained using the ALEX^2^ multiplex platform. Negative sIgE levels for apple molecular components (Mal d 1, Mal d 2, Mal d 3), as well as banana and mango extracts.

**Figure 3:**
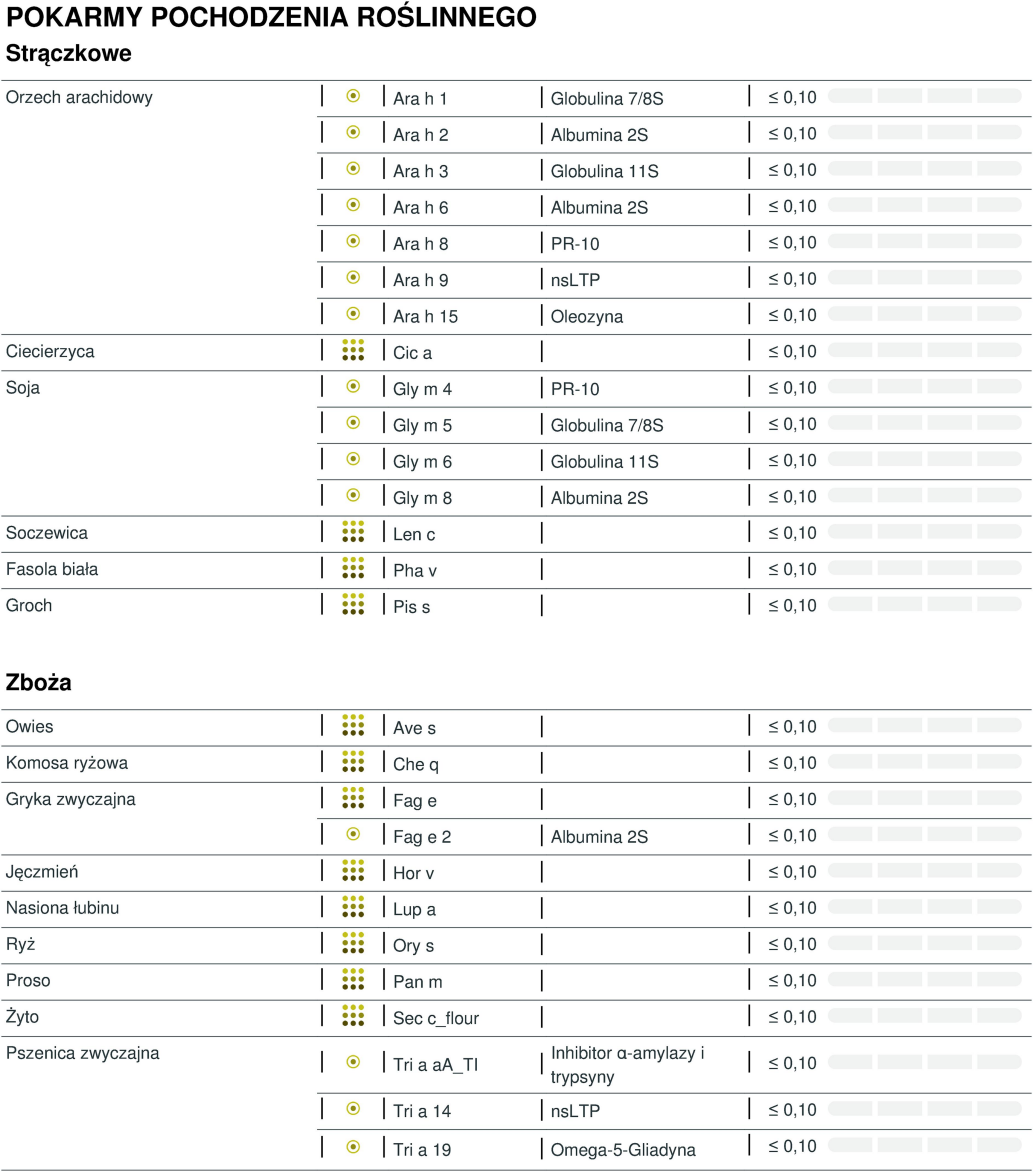
Component-resolved diagnostic results obtained using the ALEX^2^ multiplex platform. Negative sIgE levels for wheat allergen molecules (Tri a Aa_TI, Tri a 14, Tri a 19).

At the the Department of Pulmonology and Allergology at the University Hospital in Kraków, Poland, comprehensive molecular diagnostics using the component-resolved ALEX^2^ test were performed. The patient was admitted to the Department 3 years after the first anaphylactic reaction and 1 month after the most recent episode. The results in ALEX^2^ showed no increased levels of specific IgE antibodies against food allergens of plant or animal origin, however, hypersensitivity to inhalant defensins—specifically Amb a 4 (ragweed) and Art v 3 (mugwort) was confirmed. These findings correlated with the patient's clinical history of allergic rhinitis during the weed pollen season. A value of 0.35 kUA/L is considered the positivity threshold for specific IgE; however, lower levels within the range of 0.1–0.35 kUA/L may also be clinically relevant when interpreted in the context of the patient's clinical presentation and sensitization profile (1). Additionally, sensitization to inhalant- and food-derived allergen components, including nsLTPs (Art v 3, Pru p 3), PR-10 proteins (Bet v 1, Mal d 1), and profilins (Phl p 12), was evaluated, with no detectable specific IgE antibodies identified against these allergenic molecules (Figures 1–3).

Based on the mechanism of cross-reactivity between inhalant and food defensins described in the literature, a hypothesis was formulated regarding their involvement in the pathogenesis of this patient's symptoms. To verify this assumption, additional targeted diagnostic tests were performed. Clinical reactivity was assessed using native skin prick tests (SPT) with defensin-rich food products. Native SPTs evaluated after 20 min, yielded positive results for celery (5 × 5 mm), sunflower seeds (3 × 3 mm), and mango (5 × 6 mm). Following the second episode of anaphylaxis, which occurred after the consumption of herring in cream sauce with a wheat roll, additional native SPTs were carried out using the exact components of the ingested meal. The results were positive for herring in cream sauce and negative for the wheat roll.

However, considering that the consumed herring in cream sauce contained trace amounts of celery, supplementary prick-to-prick testing was performed using both raw and cooked herring. Negative results for both forms of fish indicated that the celery component present in the sauce was the most likely eliciting factor.

Moreover, during the diagnostic in the Department of Pulmonology and Allergology work-up, laboratory investigations demonstrated persistently elevated serum tryptase (ST) levels on three separate measurements (20.20 µg/L, 20.60 µg/L, 18.80 µg/L; reference: <11.4 µg/L). This prompted clinical suspicion of mast cell activation syndrome (MCAS), either in the phenotype of hereditary *α*-tryptasemia (αHαT) or a clonal variant such as systemic mastocytosis (SM). A blood sample was obtained to evaluate the presence of the KIT D816 V mutation, which was subsequently confirmed.

Diamine oxidase (DAO) activity was assessed in two independent measurements, yielding values of 8.92 U/mL and 5.34 U/mL, corresponding to an indeterminate range. In addition, prolonged persistence of the histamine-induced wheal was observed during skin prick testing, with a positive histamine response still present after 50 min.

The patient was prescribed high-dose antihistamine therapy consisting of bilastine at four times the standard dose (2 × 40 mg daily). Additionally, a low-histamine diet was implemented, along with strict elimination of defensin-containing plant foods, specifically celery, mango, peanuts, sunflower seeds, and chestnuts. Both the patient and his family received comprehensive education on anaphylaxis management, focusing on early symptom recognition and the critical importance of immediate adrenaline self-administration. The patient was prescribed two adrenaline auto-injectors. Since initiation of this management plan, no further episodes of anaphylaxis have been reported.

## Discussion

Allergy to celery is among the most common food allergies in Europe. In Northern Europe, its prevalence reaches approximately 0.45% in the adult population. Among the major celery allergens, according to the World Health Organization (WHO)/ International Union of Immunological Societies (IUIS) classification, the following are distinguished: Api g 1, Api g 2, Api g 3, Api g 4, Api g 5, Api g 6, and the increasingly described Api g 7 (2). In patients allergic to Api g 7, coexisting allergy to Art v 3 is often observed, due to the structural similarity of these components (3).

The presented case highlights the potentially crucial role of defensins as key allergens responsible for cross-reactivity between inhalant allergens, such as Art v 3 (mugwort) or Amb a 4 (ragweed), and various food allergens (2–4). In recent years, an increasing number of studies have highlighted the clinical significance of defensin-related food allergies. Although these are typically associated with a milder clinical course, they can occasionally trigger severe anaphylactic reactions (5). In the case described herein, the clinical course was particularly severe, with each episode meeting the criteria for grade IV anaphylaxis according to the classification by Błażowski et al. (6). ALEX^2^ testing was performed; however the panel did not include the Api p 7 (defensin) allergenic molecule. Therefore, considering the positive result for inhalant defensins and the known cross-reactivity, native skin prick tesing with defensin-rich foods was performed. Positive results were obtained for defensin-rich foods, including celery, sunflower seeds and mango.

During the first episode, the patient had consumed a mousse containing mango, apple, banana, and wheat. Apple, banana, and wheat had been tolerated and remain well toleranted at present, therefore, native SPTs with apple and banana was not performed. Nevertheless, native SPTs with wheat was conducted and yielded negative result. During the evaluation of the second anaphylactic reaction, the results of native SPT were positive for herring in cream sauce. However the consumed herring in cream sauce contained trace amounts of celery. Native SPTs for both fresh and cooked herring were negative. Overall, the findings support a diagnosis of defensin allergy.

According to the medical history, following peanut consumption, the patient reported skin flushing and conjunctival erythema. Given the potential risk of inducing an anaphylactic reaction triggered by native SPTs, testing with peanut and chestnut was not performed.

An important component of the diagnostic work-up was the measurement of ST levels. In this case, elevated ST levels (20.20 µg/L, 20.60 µg/L, and 18.80 µg/L) were found during asymptomatic periods, allowing these values to be considered basal tryptase levels. Notably, ST levels were not assessed during any of the previous anaphylactic episodes in the ED. The absence of serum tryptase measurement in the emergency department may reflect limited adherence to anaphylaxis diagnostic recommendations in routine emergency care settings, where priority is given to patient stabilization (7). Lack of acute-phase measurement significantly limits the ability to evaluate transient tryptase elevation during reactions, which is a key diagnostic marker of mast cell activation. According to current literature, a basal ST level exceeding 20 ng/mL is a primary indication for advanced diagnostic evaluation of mast cell-related disorders, including SM and hereditary alpha-tryptasemia (8).

Patients with MCAS may present with a broad spectrum of clinical manifestations involving multiple organ systems, including cutaneous, respiratory, gastrointestinal, cardiovascular, and neurological systems (9, 10). Consequently, the overlapping clinical manifestations across these systems significantly complicate the diagnostic process, underscoring the importance of a thorough medical history and a personalized diagnostic approach. Notably, a diagnosis of MCAS requires, among other criteria, documentation of a transient tryptase elevation (≥ 20% of baseline + 2 ng/mL) during symptomatic episodes—a requirement that could not be met in this case due to the lack of acute-phase measurements (9). Scientific data indicate that while the majority of patients with elevated basal ST levels (>20 ng/mL) are diagnosed with HαT, a smaller proportion—approximately 14%—is found to have SM (8). Molecular analysis identified the pathogenic KIT D816 V mutation with a variant allele frequency of 0.05%. According to WHO diagnostic criteria, a diagnosis of SM requires fulfillment of either one major and one minor criterion, or at least three minor criteria (10). In the current case, two minor criteria for systemic mastocytosis were fulfilled: the presence of the KIT D816 V mutation and a persistently elevated basal serum tryptase level (9, 10).

At the current stage of the diagnostic work-up, there is a high probability of an underlying clonal mast cell disorder, including indolent systemic mastocytosis (ISM) or monoclonal mast cell activation syndrome (MMAS). Measurement of serum tryptase during the acute phase of an anaphylactic reaction is of major diagnostic importance; however, due to the lack of tryptase level during the anaphylactic episodes, a diagnosis of mast cell activation syndrome (MCAS), including MMAS, cannot currently be confirmed. Given the clinical suspicion of mast cell disease, the patient was referred for further hematological evaluation, including bone marrow biopsy, at the Department of Hematology, University Hospital in Kraków.

In addition, to exclude hereditary alpha-tryptasemia (αHαT), copy number variation analysis of the TPSAB1 gene has been planned.

Clinical consideration may be concomitant histamine intolerance, although the patient's DAO activity remained within the indeterminate range, with two independent measurements between 3 and 10 U/mL. The Histamine 50 (H50) test was performed as a supportive diagnostic approach; however, its clinical utility in the diagnosis of histamine intolerance remains uncertain and has not yet been sufficiently validated (11).

Notably, one of the anaphylactic episodes occurred following consumption of herring in cream sauce with celery. It may therefore be hypothesized that the reaction resulted from a synergistic effect of multiple triggers: the IgE-mediated allergy to defensins in celery and the high histamine content of the herring (12). Symptoms of histamine intolerance and mast cell disorders frequently overlap and may exacerbate each other, contributing to a complex clinical presentation (13).

## Conclusions

In conclusion, this case highlights the requiring of a multifaceted diagnostic approach in patients with severe, recurrent anaphylactic reactions. It underscores the importance of considering less common underlying mechanisms, including IgE-mediated allergy to defensins, histamine intolerance, and primary mast cell activation disorders. Furthermore, this case emphasizes the critical clinical value of measuring ST levels during the acute phase of a reaction. Timely assessment is essential for differentiating between transient activation and underlying proliferative disorders, thereby guiding subsequent diagnostic and therapeutic strategies.

The patient was referred for further hematological evaluation for suspected systemic mastocytosis (SM), including a scheduled bone marrow biopsy. In addition, TPSAB1 copy number variation analysis is planned to exclude hereditary alpha-tryptasemia (αHαT). Future allergological evaluation may also include expanded molecular diagnostics, such as the ALEX 3 platform incorporating Api g 7 (celery defensin).

## Data Availability

The original contributions presented in the study are included in the article/Supplementary Material, further inquiries can be directed to the corresponding author.
